# A database seed for a community-driven material intensity research platform

**DOI:** 10.1038/s41597-019-0021-x

**Published:** 2019-04-09

**Authors:** Niko Heeren, Tomer Fishman

**Affiliations:** 1 School of Forestry & Environmental Studies, Yale University, New Haven, Connecticut 06511 USA; 2 School of Sustainability, IDC Herzliya, Herzliya, Israel

**Keywords:** Civil engineering, Environmental impact, Structural materials

## Abstract

The data record contains Material Intensity data for buildings (MI). MI coefficients are often used for different types of analysis of socio-economic systems and in particular for environmental assessments. Until now, MI values were compiled and reported ad-hoc with few cross-study comparisons. We extracted and converted more than 300 material intensity data points from 33 studies and provide the results in a comprehensive and harmonized database. Material intensity is reported as kilograms per gross floor area for 32 materials as primary data points. Furthermore, we augmented the data with secondary attributes for regional information, such as climate and socioeconomic indicators. The data are hosted on the version control platform GitHub using accessible data formats and providing detailed contribution guidelines. This “database seed” facilitates data analysis, accessibility, and future data contributions by the research community. In the Technical Validation we illustrate that consistency of the data and opportunities for further analysis. This database can serve scientists from various disciplines as a benchmark to determine typical ranges and identify outliers.

## Abstract (summary)

**Table:** 

Design Type(s)	data integration objective
Measurement Type(s)	material properties
Technology Type(s)	digital curation
Factor Type(s)	geographic location • temporal_interval
Sample Characteristic(s)	anthropogenic environment

Machine-accessible metadata file describing the reported data (ISA-Tab format)

## Background & Summary

Construction materials, including metals such as steel, copper, and aluminum, and non-metallic minerals such as concrete and ceramics, are responsible for around 40% of annual global resource flows^1,2^. Billions of tons of construction materials have accumulated as in-use physical stocks of buildings and infrastructure over the last century^3^, resulting in considerable economic flows^4^, resource extraction^2,5^, and environmental impacts^6^.

Quantifying material stocks and flows has been an ongoing challenge because the masses of construction materials are inconsistently measured and reported in statistics. Different methods can be used to estimate flows and stocks^7,8^. A typical approach is to use a product or service unit, such as floor area or monetary value, as a proxy for the inventory of in-use materials. It is then possible to estimate the total mass by multiplying the inventory with a known ratio of material mass per unit of inventory (see Equation 1). These ratios are often termed material intensity coefficients (MI) and can be obtained in different ways. Examples include on-site measurements^9^, construction blueprints and documents^9^, company data^10^, chemical process equations^5^, governmental assessments^11^, and construction codes and standards^12,13^. In practice, a combination of these approaches is often necessary because of the scarcity of data and to reduce uncertainties. Equation 1 illustrates the overall concept to determine the total stock *MS* of material *m* in end-use *i* at time *t*, as a product of the inventory *INV* times the material intensity coefficient.

1 $$M{S}_{m,i}[t]=IN{V}_{i}[t]\times M{I}_{m,i}[t]$$

For instance, Equation 1 can be used to determine a country’s aggregates stored in buildings by multiplying the total floor area with MI. This equation has been widely used in Material Flow Analysis (MFA)^14–16^. However, the concept of intensities has also been used in Input-Output analyses^17^ and Life Cycle Assessment (LCA)^18^ models. In the case of construction materials, the basis for analysis is often the mass unit, since conversion factors mostly exist on a mass basis, such as market price or life-cycle impacts.

Our contribution consists of compiling a database with 301 data entries for material intensities of various types of buildings using *kilograms per square meter of gross floor area (kg/m*^*2*^*)* as the unit^19^. This database was compiled with three key goals in mind:

1. Harmonization, centralization, and validation of data. Although material intensity is important for various types of assessments, data are only sparsely available and only for limited regions. Furthermore, MI coefficients are often calculated ad-hoc for a specific research, and reported precariously in assorted tables and figures. No harmonized datasets existed prior. This database collects and harmonizes the various data and may help researchers to check the plausibility of their own or other people’s work. The Technical Validation section provides insights on how to identify realistic ranges and detect outliers. We present an in-depth statistical analysis of the data, discussing regional, temporal, and other patterns in the data.
2. Accessibility to multiple disciplines. The dataset can be readily used for Industrial Ecology research, in particular MFA and LCA^20^. However, the data repository is agnostic to scientific disciplines and software, enabling other disciplines to easily access and use the data with their tools and models. Possible applications include earthquake engineering and disaster management to predict susceptibility and costs of natural disasters on buildings; for waste management, material inventories can be used to predict regional material and waste flows, as illustrated by Heeren and Hellweg^21^; and for urban studies by including the physical materials layer of cities. We also intend to use the dataset for creating synthetic building data for a global study of greenhouse gas reduction potentials by means of resource efficiency strategies^22^ (United Nations International Resource Panel, Resource Efficiency & Climate Change mitigation project, https://cie.research.yale.edu/project_main/resource-efficiency-climate-change).
3. Seed for extension. This data contribution is freely available and intended to be a seed dataset, meaning that others are invited to contribute new datasets. The database is released on a version control platform (Github) facilitating the validation of data and encourage the scientific community to engage. It allows edits, data additions, and other types of enhancements in a verifiable manner (i.e. by means of line-by-line comparisons/diff views). Therefore, the database and this article also describes procedures on how to extend the database with new data. The dataset described here reflects the initial archived release version 1.0.2.

By publishing open data, we intend to increase reproducibility of scientific publications and facilitate cumulative research^23^. It therefore fills a gap in conjuncture with other efforts such as deQo (https://www.carbondeqo.com) and Madaster (https://www.madaster.com/en) whose data are not openly available for various reasons, such as intellectual property rights. In the future, the possibility of harmonization and data exchanges between them should be investigated.

## Methods

The database consists of data that were mostly extracted from published literature. Our scoping of relevant datasets emanated from several key publications of the field of Industrial Ecology, which explains why the largest number of studies occurred in the Journal of Industrial Ecology (see also Technical Validation). We expanded our surveying efforts through citation and reference reviews, and by using the academic search tools Scopus, Google Scholar, and the search functions of the following peer reviewed journals’ homepages: Journal of Industrial Ecology, Resources, Conservation & Recycling, Building Research & Information, Journal of Cleaner Production, and Energy and Buildings. Backwards and forwards mapping were performed. The keywords used are: “material intensity”, “building material stock”, “building material”, “building stock”, “construction & demolition waste”, “construction material stocks and flows”, “housing stock”, “urban metabolism”, “mass flows”, “material composition indicators”. “material flow analysis”, “material stock analysis”, “urban mining”, and “building material inventory”.

The database^19^ contains data attributes that we term primary and secondary. Primary attributes are data that were extracted directly from the individual publications^7,10–13,21,24–50^. This includes the following non-exclusive classifications: country, city, and construction period. That means there can be multiple entries for the same classifiers. Construction period denotes a period of time the dataset authors consider a certain MI to be representative for. The material data attributes include 32 material categories. These categories reflect the ones in the data sources. For instance, if one source reported concrete as a single category but another reported concrete’s constituents in separate categories (i.e. cement and aggregate), we used the source’s sub-categories rather than attempt to convert one to the other. Another consequence is that for every data there may be empty values for material categories not reported. We discuss such issues in detail in the data consistency section. Table 1 illustrates the individual categories along with the aggregation levels that we chose for analysis described in the Technical Validation section. Material is given as intensity values in kilogram per square meter of gross floor area. It is important to emphasize that the reference area is material per gross floor area (including walls and secondary space use, such as storage space or bathrooms, across all stories) and *not* per building footprint area (i.e. the surface area a building occupies on the ground). The system boundaries of the data sources were kept true to the originals, so entries may include differing building elements, such as foundations, decorations, non-structural elements, etc. However, as some of the studies reported different types of values e.g. material per net floor area, it was necessary to convert some of the data to match our standard unit. Apart from such unit conversions, the reported data were not changed. This database seed intends to document data as true to the original publications as possible. It therefore includes some outlying data points with extreme values that may be erroneous. This is intentional – quality control of the database is “community-correcting”, meaning that data contributors and users will be able to identify suspected errors in data entry and report them, explore their causes, and correct them as necessary. Contributors of new data will be able to benchmark their numbers to those already in the database prior to publication of their research. Users of the database may filter or change data points in their private database fork as they see fit to avoid potential effects of outliers.

**Table 1:** Materials in the database and the categories used for aggregation.

Category/Aggregation				Material	No. of data points
Total	Total w/o other materials	Bio-based		Wood	278
				Paper/Cardboard	5
				Straw	0
		Metals		Steel	228
				Copper	72
				Aluminum	105
				Other/unspecified metal	95
		Construction mineral	Concrete, cement & aggregate	Concrete	257
				Cement	65
				Aggregate (gravel, sand, slag)	135
			Other construction material	Brick	184
				Mortar/Plaster	146
				Mineral fill	90
				Plaster boards/gypsum	101
				Adobe	1
				Asphalt	21
				Bitumen	73
				Natural Stone	46
				Cement asbestos sheet	32
				Clay	8
				Siding (unspecified material)	3
	Other materials			Ceramics	103
				Glass	207
				Plastics	72
				Polystyrene	30
				PVC	20
				Lineoleum	43
				Carpet	3
				Heraklith	21
				Mineral Wool	110
				Insulation (unspecified material)	29
				Other (unspecified material)	81

The right-hand side lists the materials along with the number of observations for the attribute. The left-hand side illustrates the aggregation. ‘Bio-based’ is a term used in the recent literature to describe construction materials of plant-based origin.

Further primary attributes and metadata include: author, building code, number of floors, occupation type, building type, type of data measurement, urban or rural attribute, type of floor area reported, data source authors, year of publication, publication title, publication outlet, DOI, URL, and three fields to document data aggregation, conversion, and for miscellaneous comments. Ref.^19^ also contains a codebook that describes each parameter in detail. See also section Usage Notes.

**Fig. 1:**
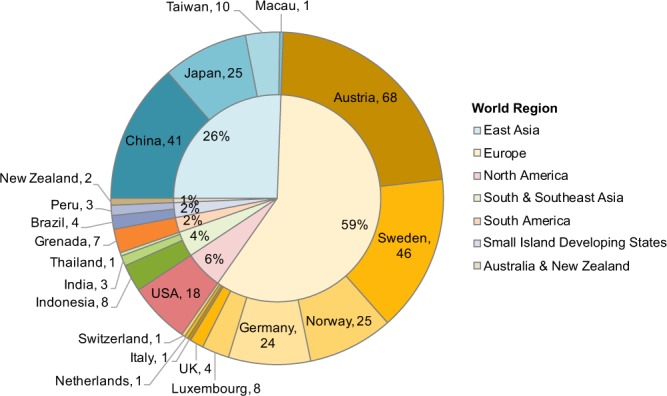
Number of data points per country. The outer pie chart illustrates the number of observations by country and the inner pie chart gives percentages by world region. The legend of world regions to the right refers to the inner circle.

The secondary data attributes were added from external sources, i.e. not directly from the publication in question^51–55^. Their purpose is to provide cross-sectional context and facilitate further data analysis. The data attributes are as follows:

- Name of the global region. Allows to cluster data as illustrated in Fig. 1.
- Köppen climate classification^53^ is a commonly used classification system differentiating the world into five main groups tropical, dry, temperate, continental, and polar, as well as further subgroups, such as semi-arid, monsoon, etc. This allows to analyze MI data for geo-spatial differences other than political or geographical region. Data were extracted from using the maps in the Supplementary Information of Peel *et al*.^53^.
- Mean distance to equator. Data were extracted by using a web tool (https://www.distance.to/).
- Heating degree days^51,56^ (https://www.kapsarc.org/research/projects/global-degree-days-database/). An indicator describing regional climates and commonly used to estimate heating energy demand. https://en.wikipedia.org/wiki/Heating_degree_day. Data were extracted from Appendix A in Atalla *et al*.^56^.
- Cooling degree days^51,56^. Similar as above, but used to determine cooling energy demand. Data were extracted from Appendix A in Atalla *et al*.^56^.
- Country land area. Source: US Intelligence Central Agency. The World Factbook 2018. https://www.cia.gov/library/publications/the-world-factbook/index.html. Data were extracted from the following table: https://www.cia.gov/library/publications/the-world-factbook/fields/2147.html
- Population^54^ (https://www.rug.nl/ggdc/html_publications/memorandum/gd174.pdf). Data were extracted from the Excel file: https://www.rug.nl/ggdc/historicaldevelopment/maddison/releases/maddison-project-database-2018
- Urbanization rate. An indicator for the percentage of population living in urban areas as opposed to rural areas. Source: United Nations Department of Economic and Social Affairs. World Urbanization Prospects: The 2018 Revision, Online Edition. (2018). https://population.un.org/wup/Download/. Table: WUP2018-F02-Proportion_Urban.xls
- GDP^54^. Gross Domestic Product being a common indicator for a country’s economic performance. Data were extracted from the Excel file: https://www.rug.nl/ggdc/historicaldevelopment/maddison/releases/maddison-project-database-2018

All primary data attributes are mandatory, including full references. Secondary data attributes, as well as BibTeX code for the reference, are optional contributions. Our aim by differentiating between primary and secondary attributes, is to keep the effort required for data contributions as low as possible, while ensuring a database consistency and quality. Other users that may require secondary attributes can collect those with relatively low effort, and, hopefully, contribute them back to the database. Please refer to the section Usage Notes and the codebook in ref.^19^ for more details.

There are a few exceptions concerning the origin of data. Heeren and Hellweg^21^ did not publish material intensities, rather they used a bottom-up GIS model to determine the national material stock for Switzerland in 2015. The data that are published here represent a national average of all Swiss residential buildings which was extracted from the geo-spatial database documented in the publication. Kleemann *et al*.^35^ published their data as material mass per volume (kg/m^3^). We approached the authors and they provided us with the building volume and floor area of the individual cases, which allowed us to convert the data to MI as defined here.

These examples illustrate the wide variety of data measurement types that are encountered. Some studies represent case studies that investigated an individual (representative) building, others report averages of several representative buildings, and others still, such as Reyna and Chester^11^, provide a national weighed average of all residential buildings. Therefore, the type of measurement is documented in a dedicated column (‘measurement_type’). Several studies could not be added because conversions to mass per gross floor area were not readily feasible, such as Schebek *et al*. 2017 and Stephan and Athanassiadis 2017^57,58^.

Most data were extracted manually. In general, it is also possible to use software such as WebPlotDigitizer to extract data points (especially for figures). In the future, users are encouraged to document data extraction in the Liberated Data Project (https://github.com/nheeren/liberated_data), which makes it possible to verify the procedure.

We extracted all available material categories from the studies in our survey. In some cases, materials were assigned or summed into a generic ‘other’ category. In other cases, studies already contained a generic category or the actual materials were not further specified, such as “roof covering”, and we adopted these to the generic ‘other’ category as well. The database contains a dedicated comment variable (‘comment_aggregation’) to document such types of data aggregation or categorization.

Several actions ensure that data are accessible to researchers from different fields using different types of software. We have chosen a version controlled repository as the platform to host the data and a comma-separated values file (CSV) using the RFC 4180 specification^59^. This allows for easy data contributions, either by pull requests or manual additions via the Github web interface (https://help.github.com/articles/about-pull-requests/). The changes in the database can be conveniently tracked by diff views. CSV is a portable and agnostic format commonly used in data science software and most types of spreadsheet software, such as LibreOffice Calc or Microsoft Excel, can process it. Moreover, we provide extensive explanation on the data in a codebook, which avoids ambiguities when interpreting and parsing the data. Please refer to the section “Usage Notes” and the documentation in ref.^19^ for more details on the encoding of the data and the data contribution process.

## Data Records

All data are available on Zenodo in ref.^19^. It reflects the release version 1.0.2 of the Github repository https://github.com/nheeren/material_intensity_db. The Github platform was chosen to facilitate growth of the database in terms of features, such as new attributes or parsing and analysis scripts, as well as new datasets from existing and new literature.

The repository contains the following files:

1. *README.md* is a text file, using markdown syntax^60^, describing the repository and its contents.
2. *README.pdf*: A rendered version of the *README.md* markdown file.
3. *buildings.csv* is a RFC 4180^59^ formatted CSV file that contains the material intensity data with one dataset per line.
4. *codebook.md* is a text file using markdown syntax^60^. It gives an exact explanation of the columns’ meaning in the actual codebook (*codebook.csv*). It also provides instructions and definitions, such as the attributes to use for missing values.
5. *codebook.csv* is a RFC 4180^59^ formatted CSV file that defines for every column in the data file how it is to be formatted, documented and what type of data are allowable and required.
6. *codebook.pdf*: A rendered version of the *codebook.md* markdown file.
7. *CONTRIBUTING.md* is a text file using markdown syntax^60^ and explains the procedures of contributing new datasets or database parameters or correcting existing datasets.
8. *CONTRIBUTING.pdf*: A rendered version of the *CONTRIBUTING.md* markdown file.
9. *buildings.bib* is a text file that holds the references for all data records in the BibTeX format (http://www.bibtex.org/Format/).
10. *special_values.md*: Flowchart as used in *codebook.md* using the mermaid syntax (https://mermaidjs.github.io).
11. *special_values.png*: Rendered version of the flowchart in special_values.md.

The codebook represents an essential part of the database. It defines how data are formatted and how missing values were treated. This helps researchers to understand and use the data for their own analysis. Moreover, it clearly defines how new contributions need to be formatted.

The primary data attributes, i.e. publication metadata and material intensity data, are extracted from the individual publications^7,10–13,21,24–50^. The secondary attributes, i.e. additional regional data, such as climate information, are extracted from further sources (see Methods section). See the Methods section and the codebook in ref.^19^ for more information on data provenance and the individual attributes.

## Technical Validation

### Data overview

The data consist of 301 datasets documented in 33 publications^7,10–13,21,24–50^. Table 2 illustrates the different sources. The number of data points per study varies widely. More than half of the data originate from only three publications, and 90% of the data are found in half (i.e. 16) of the publications. Almost all of the studies (31) are peer-reviewed journal publications, one of which was in press at the time of writing (Symmes *et al*.)^44^. Gruhler *et al*.^27^ is part of a German book series, Hong *et al*.^29^ is part of a conference proceedings. The publications appeared in 12 different types of outlets, with two peer-reviewed journals, the Journal of Industrial Ecology and Resources, Conservation & Recycling covering roughly half of that.

**Table 2:** Publications by outlet.

Publication	Type	Count	Percentage
Journal of Industrial Ecology	Journal	11	33%
Resources, Conservation & Recycling	Journal	7	21%
Building Research & Information	Journal	4	12%
Building and Environment	Journal	2	6%
Journal of Cleaner Production	Journal	2	6%
Energy and Buildings	Journal	2	6%
Buildings	Journal	1	3%
Ecological Economics	Journal	1	3%
Sustainability Science	Journal	1	3%
ACEEE 2014	Conference Proceedings	1	3%
IÖR Schriften 38	Book	1	3%
**Total**		**33**	**100%**

The data cover 21 countries from seven world regions (Fig. 1). 59% of the data points describe European material intensities. To our knowledge, this is one of the largest collection of material intensity data to date. Kleemann *et al*.^35^ and Ortlepp and colleagues^41^ have previously complied data from their own work and other studies.

### Data Consistency

In this section we give an overview of the data and their inherent consistency. Kleemann *et al*.^35^ is the only data source that uses measured material intensity from waste material being transported off from building demolition sites. Opposed to that, most other studies determine material inventory mathematically, i. e. by calculating the volume of building elements and multiplying it with the material density of the single layers.

We identify several factors that could contribute to inconsistencies in the data. The compiled database is unbalanced, meaning that relatively few sources created many data points while others provide only a single data point. Some studies focused on a single material while others on multiple ones. It is not always clear if different sources refer to different materials with the same term, for instance mortar, and conversely studies sometimes use the terms steel and iron ambiguously. Some studies account for metals as a generic category, and others detail different types of metals. Each study used their own method to collect and harmonize data, and the authors usually do not describe uncertainties qualitatively nor quantitatively. Furthermore, each study was conducted with different objectives in mind (e.g. waste estimation, socio-economic comparisons, disaster management, etc.). Therefore, the resolution in materials considered and their system boundaries may differ. For some sources it is unclear if their data refer to gross or net floor area. These factors also culminate in high variability in the number of observations per material category, from a single datapoint for adobe to 278 for wood.

**Fig. 2:**
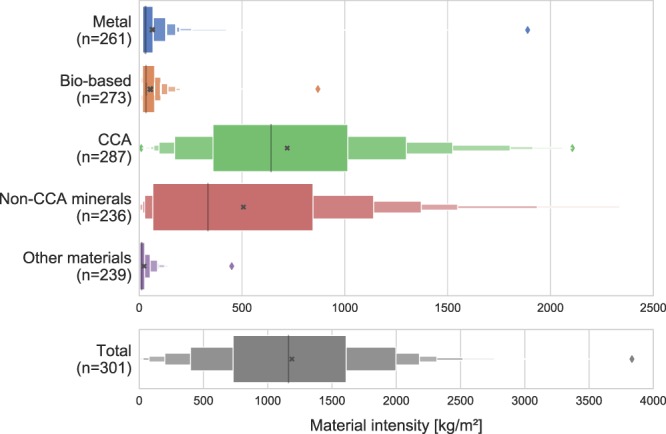
Letter-value plots of material intensity for different categories. This type of plot is very similar to box plots, but provides additional information of the data distribution, because the boxes illustrate multiple quantile ranges^62^. The largest box represents the 4-quantile range (quartiles) or 50% of the data, the second largest the 8-quantile range (octiles) or 75% of the data, and so forth. The grey lines, the cross, and the colored diamonds signify median, mean, and outliers, respectively. n denotes the number of observations for the aggregated category. The outlier for ‘Non-CCA minerals’ at 3044 is omitted in the upper plot for visual clarity.

Nevertheless, by aggregating materials into five overarching categories (as illustrated in Table 1), the values seem to converge to certain statistical patterns. On average, the material intensities of concrete, cement and aggregate (CCA) and other minerals are an order of magnitude higher than the ones for metals and bio-based materials (Fig. 2). The distributions of the four categories (metals, bio-based, CCA, and other minerals are asymmetrically skewed to the left with long right-handed tails, leading to means that are higher than the medians (Fig. 2). Metals is the most extreme case, with the mean nearly equal to the value of the 3^rd^ quartile. Interestingly, when summing the two construction minerals categories together, their distributions become more symmetrical, suggesting that the two complement each other. Because of their higher magnitudes compared with metals and bio-based materials, the two minerals categories dominate the statistics of the summed mass per square meter. We chose to proceed with analyses with the means rather than the medians, because we are interested in the effects of seemingly outlying material intensity values and the implications of the high variance in the data, which is captured by the means and their standard deviations.

**Fig. 3:**
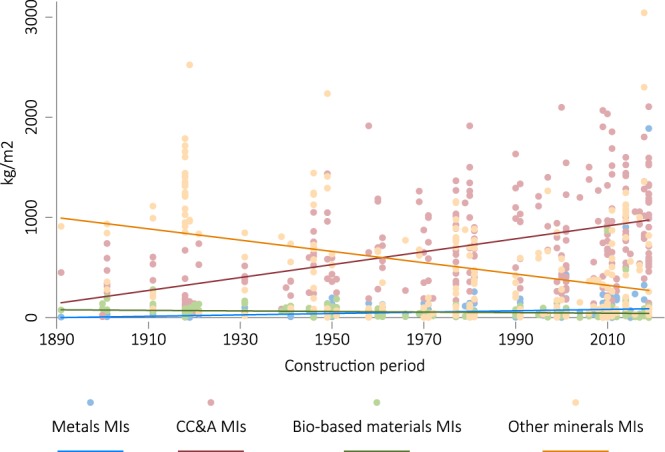
OLS regressions of categorical material intensity values. It refers to the reported upper limit of the construction period as the independent variable.

We compared the results with the deQo database (https://www.carbondeqo.com). Unfortunately, the full data for a complete statistical comparative analysis is not available through the web interface. We therefore reverted to a simple visual comparison. Selecting “Program Category” in the deQo database, the distributions seem to be in line with our Total results (cf. Fig. 3). For instance, the boxplots in deQo’s “Residential” category (n = 124) cover a range from 98 to 1882 kg/m^2^, with the median being 654 kg/m^2^. In the “Commercial” category (n = 174) the whiskers range from 224 to 2317 kg/m^2^ and the median is at 1034 kg/m^2^.

The factors that we identify above fundamentally relate to the source of each data point. We therefore examined what, if any, relation the source publication has on material intensity values. For each of the six material categories we conducted an Ordinary Least Squares (OLS) regression with material intensity as the dependent variable and the sources of the data as the independent categorical variables, equivalent to one-way ANOVA, and tested the statistical significance of the difference of the mean of the observations of each source from the grand mean, taking into account the unbalanced nature of the dataset (Table 3, refer to the Supplementary Files 1 and Supplementary File 2 for detailed procedures and numerical results, respectively using the Stata software^61^). For metals and bio-based materials, very little of the variance can be explained by the publication’s authors attribute. For the most part differences in each source’s mean MI values from the grand means are not statistically significant. In comparison, for the two non-metallic mineral MI categories and their aggregated sums category, a notable share of the variance can be explained by the publication source from which it originates, and notably some of the sources of the data have statistically significant mean MIs that are different from the grand mean. Similar effects are observed in the *all materials* category: Because the material intensities of minerals are higher by one order of magnitude than metals and bio-based materials, their amounts dominate the aggregated category’s results.

**Table 3:** ANOVA results testing whether the source publication has an effect on mean material intensity values.

Material	No. of obs.	ANOVA F (Prob > F)	r^2^	significant at 10%	significant at 5%	significant at 1%
**metals**	261	0.0827	0.1514	0	0	0
**bio-based**	273	0	0.2257	2	2	2
**concrete**	287	0	0.3861	3	3	7
**other minerals**	236	0	0.4016	0	2	1
**Construction minerals**	290	0	0.6444	3	5	14
**Total**	301	0	0.6349	3	4	17

Materials are aggregated to the material categories of Table 1.

These findings illustrate the effects of the data sources on the MI values. They are important to note because for most countries we currently have only data from single publications. Only four out of the 21 countries have observations from multiple publications (Germany, Japan, China, and the United States). In other words, for the other 17 countries there is perfect collinearity between the country and the publication. It is therefore currently impossible to separate the idiosyncrasies that may arise due to actual country differences from the idiosyncrasies that relate to a study’s data compilation procedures and methods. Because cross-country comparisons are one of the long-term goals of this database, this current limitation calls for two actions:

1. Procurement of more data from more varied sources, to enable the allocation of attribution of variance to the source vs. the country.
2. Advocate for more harmonized data compilation methods by authors to minimize publication source-related idiosyncrasies.

### Observations

Although the current low numbers of observations of many individual material categories and high collinearity of authorship and country may be a setback for cross-country comparisons until more data are added to the database seed, it does not hamper other applications of the data. The data as-is can be used in a pooled fashion to provide new insights. We demonstrate this with an analysis of trends in MIs over time. OLS regressions of the values of MIs with the reported upper limit of the construction period as the independent variable show that between 1890 and 2018 metal MIs grew by 0.7 kg/m^2^ per year and bio-based MIs decreased −0.3 kg/m^2^ per year. CCA MIs grew 6.4 kg/m^2^ per year, while the other mineral MIs decreased by −5.6 kg/m^2^ per year. This suggests that CCA is substituting bio-based materials and other minerals, which include bricks (Fig. 3). The visualization of the data highlights the heterogeneity of construction styles and data compilation methods, yet a relatively clear trend emerges.

In the Supplementary Files 1 and 2 we present further robustness tests for this analysis, as well as for the sources-related variances, described above, and other potential issues.

This analysis is meant to provide an understanding of building material intensity as of the current database and it illustrates the consistency of the database. It exposes trends in the data and suggests that there are likely more relationships to be uncovered by further analysis.

## Usage Notes

The database contains CSV files that can be readily read by common analysis software. The associated codebook avoids ambiguities concerning data parameters. The purpose of the codebook is to also provide a clear guideline for data contributors on how data need to be formatted. Currently, we do not provide scripts to parse or convert the data. However, we intend to add such tools to the repository in the future. They will include validation routines that check the codebook against the data file, conversion tools to create other file formats, such as .xlsx files, and different types of analysis tools for Microsoft Excel, Python, and R.

The data are hosted on a version-controlled platform in order to facilitate data contributions. By creating a pull request, contributors can ask for a review of their data, which will then be performed by the project maintainers. In the future, the repository will ideally also use coverage tools that allow automated validation of data consistency by comparing the new contributions with the rules defined in the codebook.

In order to correct or contribute data, the contributor needs to familiarize themselves with the guidelines in the contributing file and the codebook. The codebook defines which parameters are mandatory and which are optional. All primary data attributes are mandatory, while secondary data attributes, as well as the BibTeX reference, are optional contributions. Database users that have an interest to use certain secondary datasets can contribute those at a later stage. The codebook also has guidelines concerning the type of data and the data classifications that should be used when providing new data.

Special emphasis lies on the definition of missing data in order to avoid any ambiguity and facilitate data analysis. It is differentiated between *no observation* and *no information*. Therefore, missing values have different notation with *NULL* referring to the former and *NA* to the latter. The database cannot contain empty values. *NULL* is the default value for empty or missing values. Only if the data contributor is certain that the data source does not contain a value the value *NA* is used. In case the data source contains a reference to the parameter in question, but does not provide a numerical value, the value *unspecified* is used. Otherwise the numerical value is used. The number *0* must only be used if it is specified or implied as such. An excerpt from the *codebook.md* file:

- *NULL*: Missing value that has *no observation*. This is the default empty value of cells in a new column or row. That means, the parameter was not evaluated by the person providing the data. For example, this is the default value if a new column is added to the database. Without revisiting the studies it is not possible to make a judgement on the values and all rows would therefore be *NULL*. The same applies if a data contributor decides not to provide the (optional) secondary data attributes – they need to be *NULL*. Ideally there should be no *NULL* valued cells in the database and contributors are encouraged to resolve *NULL* values.
- *NA*: Missing value that has *no information*. That means no data were provided, is not applicable, or could not be attributed. This implies that the data contributor looked for the data in the source, but no (suitable) value was found. An example: If a study on buildings reported only steel in reinforced concrete buildings, then the ‘concrete’ column will be *NA*, since no value for concrete is present. It is at the contributor’s discretion to calculate the concrete from the available numbers and mention the calculation steps in the comment column.
- *unspecified*: The data source contains an explicit unspecified value, such as “unspecified”, “not available”, “-”, “unknown”, “unclear”, “trace amounts”, “some”, etc. This means that the data creators considered this attribute but have not provided a numerical value (zero or non-zero number). An example: In a study on a building the data creators state that copper content is known to be part of the building in an unknown amount shall have ‘unspecified’ in the corresponding column.
- *0*: A zero value is simply maintained as the number zero (0). However, it must only be used if the number has been measured and provided in the data source. It must not be used as a placeholder for missing values.

This distinction is important to avoid ambiguities concerning the reason for missing data. Sometimes a data contributor would find data that were specifically labelled as unspecified, sometimes they would find no data (lack of information), and sometimes they did not look for data (lack of observation). The 1.0.2 version of the database contains some *NULL* values, because we added new attributes to the database after the data collection had been completed.

Concerning the data collection, the file *codebook.csv* contains clear guidelines as to what values are allowed. Contributors can perform calculations to aggregate or produce parameters. Any alteration to the original data, such as unit conversions, aggregations, etc., must be documented in one of the designated comment columns. In order to increase transparency of data extraction, we encourage the use of the Liberated Data Project (https://github.com/nheeren/liberated_data). This project aims at making data extraction from literature sources reproducible and allows researcher share data they extracted from publications.

It is also possible to propose structural database changes by means of pull requests. Such changes, however, require that the person creating the pull request enhances the CSV file and updates the codebook at the same time. This is necessary to ensure that the codebook always covers the database parameters and, in the future, ensures the functionality of the validation scripts.

In version-controlled code platforms, every change of the code or data are characterized by a unique SHA-1 commit hash. This technique allows to identify and compare every versions of the database. The data that are used in this data descriptor article, is additionally tagged as the v1.0.2 release.

Finally, in GitHub repositories it is possible to interact with a project by means of so-called issues (https://help.github.com/articles/about-issues/). At the time of writing the repository contains an issue #1, which keeps a list of potential data sources (https://github.com/nheeren/material_intensity_db/issues/1). Contributors are encouraged to extract data from these sources and provide them back to the project by means of pull requests.

## Supplementary Information

### ISA-Tab metadata file

Download metadata file

### Supplementary Information

Supplementary File 1

Supplementary File 2
