# Supplementary material for: A database seed for a community-driven material intensity research platform
Source: Sci Data. 2019 Apr 9;6:23. doi: 10.1038/s41597-019-0021-x (PMC6480936; doi:10.1038/s41597-019-0021-x)
Supplement: Supplementary file 2 — Supplementary File 1 [file 41597_2019_21_MOESM2_ESM.docx]

﻿Supplementary File 1

A Database Seed for a Community-driven Material Intensity Research Platform

Niko Heeren^1^*, Tomer Fishman^1,2^

1. School of Forestry & Environmental Studies, Yale University, New Haven, Connecticut 06511, USA.

2. School of Sustainability, IDC Herzliya, Israel

*corresponding author: Niko Heeren ([niko.heeren@yale.edu](mailto:niko.heeren@yale.edu))

Content

[Data consistency 1](#_Toc532552517)

[Observations 2](#_Toc532552518)

[References 6](#_Toc532552519)

[Table 1: F and r^2^ statistics describing the significance and goodness-of-fit of the models 2](#_Toc532552173)

[Table 2: Detailed model results of all six MI families 3](#_Toc532552174)

[Figure 1: Diagnostic post-estimation plots of all six models 5](#_Toc532552162)

This document contains additional information on the data Technical Validation, presented in the main article.

# Data consistency

To test the effects of publication source on the values of material intensity categories (aggregated as described in Table 1 of the data descriptor), we conducted an Ordinary Least Squares (OLS) regression, with material intensity as the dependent variable and the sources of the data as the independent categorical (factor) variables. This approach is computationally equivalent to a one-way ANOVA. We ran this model for each of the six material intensity families, independently of each other, using the following model:

$$MI=\beta_{0}+\beta_{1}source+\epsilon$$

$MI$ is the material intensity, $source$ is a category (factor) variable describing the 31 publications, $\beta_{0}$ and $\beta_{1}$ are the regression coefficients to be fitted ($\beta_{0}$ can be interpreted as the mean of the base category and $\beta_{1}$ is the difference of each other $source$ category from the base category), and $\epsilon$ is the unexplained residual. The models were fitted in Stata 14 using the *regression* command.^1^

The resulting F and r^2^ statistics describe the significance and goodness-of-fit of these models. The model for metal MIs which has a significance level of 8%, and the other models are highly significant beyond the 1% level. Little of the variance of metal MIs and bio-based MIs can be explained by their sources, as indicated by their smaller r^2^ values, whereas for each of the disaggregated minerals categories (CCA MIs and other minerals MIs) around 40% of the variance is explained by the source. The highly aggregated sum of minerals and all material MI categories have relatively high r^2^ values, suggesting that much of the variance in MI values can be explained merely by the source.

Table 1: F and r^2^ statistics describing the significance and goodness-of-fit of the models

|  | **Number of**  **observations** | **F** | **Prob > F** | **r^2^** |
| --- | --- | --- | --- | --- |
| **Metal MIs** | 261 | 1.42 | 0.0827 | 0.1514 |
| **Bio-based MIs** | 273 | 2.66 | 0 | 0.2257 |
| **CC&A MIs** | 287 | 5.41 | 0 | 0.3861 |
| **Other minerals MIs** | 236 | 7.62 | 0 | 0.4016 |
| **Sum minerals MIs** | 290 | 17.48 | 0 | 0.6444 |
| **Overall MIs** | 301 | 15.84 | 0 | 0.6349 |

To further analyze the effects of publication source on the values of MIs, the mean MIs of each material family in each publication source were analytically compared to the grand mean. The difference of the categorical means (i.e. each source’s mean) was tested for statistical significance, as post-estimation tests of the regression results described above. We used Stata 14’s *contrast* command with the *gw* operator for observation-weighted means, to account for the unbalanced nature of the dataset.^1^

We observe that for metal MIs, despite some high-valued differences from the grand mean for several publications, only the mean MIs from three publications have statistically significant differences from the grand mean at the 1% level (four publications at the 10% level). Similarly, bio-based MIs also have only four publications significantly different at 1% (six at 10%). CC&A is markedly different with 8 (15 at 10%), and other minerals MIs with 6 (10 at 10%). The sum minerals MIs means of 15, or half, of the publications are statistically significantly different from their grand means (18 publications at 10%) and likewise for 16 out of the 31 publications (20 at 10%).

It should be noted that the calculated standard errors are in part functions of the number of observations per publication source. This means that similarly different mean MIs can have different statistical significance. For example, the difference from the grand mean of the mean metals MIs of Kleemann et al. and Condeixa et al. are similar (-41.01 and -42.62, respectively) but Condeixa et al.’s mean is calculated from 4 MIs and thus this mean is treated as more uncertain than Kleemann et al.’s 68 MIs. This observation suggests that pairwise comparisons of all MIs by source may reveal further insights, but may not be analytically informative and in any case are beyond the scope of this study.

The full results of these comparisons are presented in the file *SI2.xlsx*.

# Observations

To analyze the change in MI values over time, we conducted OLS regressions for each MI family with the following model:

$$MI=\beta_{0}+\beta_{1}year+\epsilon$$

$MI$ is the material intensity, $year$ is the vintaging period end year, $\beta_{0}$ and $\beta_{1}$ are the regression coefficients to be fitted, and $\epsilon$ is the unexplained residual. The detailed model results of all six MI families are reported in Table SI-2.

Table 2: Detailed model results of all six MI families

|  |  | **Coefficient** | **S.E.** | **P value** | **r^2^** | **No. of obs.** |
| --- | --- | --- | --- | --- | --- | --- |
| Metals MI | year $\beta_{1}$ | 0.69 | (0.24) | 0.005 | 0.03 | 261 |
|  | Constant $\beta_{0}$ | -1297.44 | (478.41) | 0.007 |  |  |
|  | Model F | 8.1 |  | 0.005 |  |  |
| Bio-based MI | year $\beta_{1}$ | -0.28 | (0.12) | 0.019 | 0.02 | 273 |
|  | Constant $\beta_{0}$ | 616.45 | (237.59) | 0.01 |  |  |
|  | Model F | 5.61 |  | 0.02 |  |  |
| CC&A MI | year $\beta_{1}$ | 6.45 | (0.68) | 0 | 0.24 | 287 |
|  | Constant $\beta_{0}$ | -12043.6 | (1353.44) | 0 |  |  |
|  | Model F | 88.96 |  | 0 |  |  |
| Other minerals MI | year $\beta_{1}$ | -5.64 | (0.9) | 0 | 0.14 | 236 |
|  | Constant $\beta_{0}$ | 11657.92 | (1782.15) | 0 |  |  |
|  | Model F | 39.16 |  | 0 |  |  |
| Mineral sum MI | year $\beta_{1}$ | 1.23 | (1.02) | 0.228 | 0.005 | 290 |
|  | Constant $\beta_{0}$ | -1304.46 | (2011.76) | 0.517 |  |  |
|  | Model F | 1.46 |  | 0.23 |  |  |
| All materials MI | year $\beta_{1}$ | 0.42 | (1.04) | 0.686 | 0.0005 | 301 |
|  | Constant $\beta_{0}$ | 354.89 | (2062.17) | 0.863 |  |  |
|  | Model F | 0.16 |  | 0.69 |  |  |

The results show that the overall explanatory power of the models are low (very low r2 values). However, for Metal MIs, Bio-based MIs, CC&A MIs, and Other minerals MIs, the models are statistically significant (model F statistics with significant P values).

This is in contrast with the mineral sums MI and all materials MI whose F statistics are not significant, signifying that for these two MI families, this model is not informative. This can also be seen in their $\beta_{1}$ values, which are not significantly different from zero, suggesting that the overall MIs of minerals and of all materials have not changed over time. This should not be surprising, because the coefficients of the two minerals sub-categories (CC&A and Other minerals) were found to nearly cancel each other out, as described above.

Diagnostic post-estimation plots of the six models are presented in the Figure SI-1 below.

| **Model** | **Residual-vs-fitted plot** | **Q-Q plot of residuals vs. normal distribution** | **Histogram of residuals** |
| --- | --- | --- | --- |
| Metals MI |  |  |  |
| Bio-based MI |  |  |  |
| CC&A MI |  |  |  |
| Other minerals MI |  |  |  |
| Sum minerals MI |  |  |  |
| All materials MI |  |  |  |

Figure 1: Diagnostic post-estimation plots of all six models

# References

1. StataCorp. *Stata Base Reference Manual. Release 14*. (Stata Press, 2013).
